# Socio-Demographic and General Health Factors Associated with Quality of Life in Long-Term Breast Cancer Survivors from Southwestern Poland

**DOI:** 10.3390/ijerph18179321

**Published:** 2021-09-03

**Authors:** Małgorzata Socha, Krzysztof A. Sobiech

**Affiliations:** Department of Human Biology, University School of Physical Education in Wrocław, al. I. J. Paderewskiego 35, 51-612 Wrocław, Poland; krzysztof.sobiech@awf.wroc.pl

**Keywords:** breast cancer, quality of life, comorbidity, abdominal obesity, mental health, physical functioning

## Abstract

*Background*: Identification of factors associated with quality of life (QOL) in breast cancer survivors can contribute to better functioning in this group of patients. The study aimed to assess the association between QOL and anthropometric, sociodemographic, and medical characteristics in postmastectomy women from southwestern Poland, 9.4 (±6.5) years after completed treatment. *Materials and methods*: QOL was estimated with the SF-36v2 questionnaire in 250 survivors aged 62.8 (±8.0) years with previously histologically confirmed invasive breast cancer. *Results*: Women in this study rated their overall QOL with an average of 60.7 (±17.9), Mental Component Summary of 62.8 (±19.8), and Physical Component Summary of 57.5 (±18.8) points. The use of multivariate regression analysis revealed that depression, chronic diseases, abdominal obesity, and pregnancy history have a statistically significant negative effect on the QOL of women after mastectomy, whereas participation in regular physical activity, living with a partner, the education level ≥ 12 years, and living in the city were associated with a higher QOL assessment. There were no significant relationships between QOL and the age, time since surgery, type of treatment, smoking, and occupational status of the patients. *Conclusions*: Health education, greater social support, specialist care in the treatment of comorbidities, and propagation of a physically active lifestyle can improve the physical and mental functioning of breast cancer survivors long after diagnosis and treatment.

## 1. Introduction

Advancements in breast cancer diagnosis and treatment have increased the survival rate of patients. In this context, the study of determinants of quality of life (QOL) is an important and topical problem for breast cancer patients. According to Sert et al. [1], only diagnosis of breast cancer is an independent negative determinant of QOL, mostly in the component of social functioning. Authors point to significant changes in QOL in women after mastectomy. QOL is lowered in a wide range of its components among breast cancer survivors, even many years after diagnosis [2,3]. Pain, fatigue, and sleeplessness are the most frequent symptoms reported by breast cancer patients, while body mass gain is a common problem after cancer treatment completion. Undesirable changes in weight and body composition in addition to the predisposition to breast cancer, especially among postmenopausal women, increase the probability of disease recurrence and death. Cancer treatment-related body mass gain can increase the risk of prevalence of comorbidities and negatively affect QOL. A higher body fat level was shown to be associated with lower QOL in breast cancer patients receiving radiotherapy [4,5] and in patients with low levels of physical activity [6]. Overweight or obese postmenopausal women with breast cancer who gained body weight after diagnosis reported the worst QOL and fatigue as compared with women who maintained a stable body weight [7]. Age, ethnic origin, residence, employment status, comorbidities, education level, and lifestyle components such as smoking or level of physical activity are significant predictors for health-related QOL status [1,8,9]. The prevalence of accompanying chronic diseases affects the survival rate among breast cancer patients due to side effects of the therapy and lowers the QOL in breast cancer survivors [8]. Obesity and sedentary lifestyle in breast cancer patients are associated with low levels of physical activity. They increase the risk of cardiovascular diseases and impair QOL [10].

In summary, QOL is based on the interaction of multiple factors, and their influence may vary between populations. The recent research results based on a database of the Central Statistical Office of Poland in the years 2000–2016 showed rising mortality trends and an increase in the number of lost years of life due to breast cancer in the female population in Poland [11]. Polish studies of the quality of life among breast cancer patients included women who had undergone surgery for breast cancer and in a short time came in for follow-up appointments to the hospital [12,13,14,15,16], but still little is known about the physical and mental health of breast cancer survivors long after the completion of therapy.

Based on the above issues, the aim of the study was to evaluate the QOL in Polish women after mastectomy long after completion of breast cancer treatment, with the use of the Medical Outcomes Study 36-Item Short Form Health Survey (SF-36).

## 2. Materials and Methods

### 2.1. Participants

Participants were members of the postmastectomy women’s clubs in southwestern Poland. The Federation of “Amazon” Associations (Polish postmastectomy women’s clubs) is a self-help, nonmedical organization supporting and giving practical help to women affected by breast cancer. Through this organization, patients after surgery and treatment in oncological hospitals receive psychological support, participate in meetings with doctors and dieticians, and take part in rehabilitation exercises. Women can join an Amazon club at any stage of cancer. Information about Amazon groups can be found at oncology hospitals. Trained volunteers (women who have survived breast cancer) visit sick patients in hospitals, provide support immediately after surgery, and invite them to join the club. Club members generally meet once a month, but they participate in several activities each week, for example, twice for rehabilitation gymnastics, once for swimming, and an additional art workshop. Amazon clubs are a place to socialize and exchange experiences about treatment methods and oncological rehabilitation, and the volunteers who work with them give women strong support.

A total of 250 long-term breast cancer survivors aged 62.8 (±8.0) years, 9.4 (±6.5) years after diagnosis, were included in this present analysis. Sample size was calculated using an internet-based calculator, resulting in a minimum of 246 patients. Request for inclusion in the research was directed to 400 patients; 303 women (76%) agreed to participate in the study, 53 of which were excluded from the study due to incomplete data. If the following criteria were met by the patients, then they were invited to participate: histologically confirmed invasive breast cancer, radical or partial mastectomy (in no case as a form of prevention), minimum 3 years after surgery and completed treatment, no recurrence of breast cancer, and no receiving adjuvant therapy at the time of the study, without any other previous or concurrent cancer (exclusion criteria). Patients who met the inclusion criteria were assessed with a specific questionnaire by one researcher. The study was approved by the Research Bioethics Committee of the University School of Physical Education in Wroclaw (Reference No. 27/2014). The invitation to participate in the study was extended to the presidents of postmastectomy clubs in southwestern Poland. The study was conducted in groups after initial consent and scheduling. All participants volunteered to take part in the study and gave their written informed consent in accordance with the Declaration of Helsinki. Women were informed in detail about how the study would be conducted and their participation in it. They could refuse to participate in the study at any time during the project.

### 2.2. Procedures

QOL was assessed with the use of a standardized 36-item self-administered Short Form Health Survey questionnaire—a valid tool of assessment of general health status in women after breast cancer therapy [17]. Permission was obtained from QualityMetric Incorporated to use the Polish version questionnaire (IQOLA SF-36v2 Standard, Poland). The SF-36 consists of 11 questions that include 36 items allowing QOL assessment in eight areas of health status: Physical Functioning (PF), Role Physical (RP), Bodily Pain (BP), General Health (GH), Vitality (VT), Social Functioning (SF), Role Emotional (RE), and Mental Health (MH). Each concept can be scored from 0 to 100, where a higher score indicated better health subjectively. The questionnaire permits a full assessment of overall QOL (OQOL), the Physical Component Summary (PCS), and Mental Component Summary (MCS). The PF, RP, BP, and GH subscales constitute the Physical Component Summary (PCS); the VT, SF, RE, and MH subscales constitute the Mental Component Summary (MCS). A supplementary survey questionnaire comprised questions about age, place of residence, education, marital status, occupation status, comorbidities, smoking, and regular physical activity, also including data on the type of undertaken surgery and breast cancer treatment methods. According to the WHO recommendations, waist circumference was measured with an anthropometric tape to the nearest 0.5 cm, at the midpoint between the lower margin of the last palpable rib and the top of the iliac crest. Normal waist circumference was considered <80 cm, and abdominal obesity was considered ≥80 cm [18].

### 2.3. Statistical Analysis

Statistica software package (version 13.3, license from StatSoft Polska, Kraków, Poland) was used to analyze the data. Student’s t-test or nonparametric Mann–Whitney U test for independent groups, one-way comparisons of variance (ANOVA) with post hoc analysis or Kruskal–Wallis test was used to compare SF-36 scores between categories of sociodemographic, anthropometric, and medical variables. Multivariate regression analysis was used for the prediction of factors affecting the QOL in the studied women. Regression coefficients were determined with the use of the method of least squares. The measure of model fitting was the coefficient of determination R^2^. The significance of the study model was verified with an F-test. We studied the relationship between QOL according to the SF36 questionnaire (dependent variable) and all potential sociodemographic and medical factors recognized in the study, which may affect QOL (independent variables). Separate models were tested for each SF-36 scale, Physical Component Summary (PCS) and Mental Component Summary (MCS), and overall QOL (OQOL). Qualitative variables were transformed into binary (0 or 1). Significant level was set at *p*-value < 0.05.

## 3. Results

All sociodemographic and general health condition characteristics of the 250 breast cancer survivors can be found in Table 1. The mean age of the participants at the time of the study was 62.8 (±8.0) years, the most numerous group were women in the age range 50–69 years. Only 5.6% of subjects were below 50 years, and 17.6% were above 50 years. In 28.4% of the participants, education level was <12 years; in other women, schooling lasted 12 years or more. Only 9.6% of respondents lived in rural areas, and other persons lived in the city (90.4%); 36% of women were living alone, and 64% were living with husband or partner. In terms of their occupational status, only 10.4% were working while 89.6% were not working; 88.2% of women had a pregnancy history. Nearly 45% of study subjects were current smokers. Mean waist circumference was 108.8 (±10.6) cm. Abdominal obesity classified by waist circumference occurred in 88.4% of subjects. The participants frequently mentioned the following comorbidities: hypertension (40.8%), arthritis (37.2%), thyroid disease (24.8%), cardiovascular disease (21.2%), and diabetes (14%); 8.8% of women had depression. During the study, 41.2% of women declared participation in regular physical activity.

The basic clinical data are shown in Table 2. The mean age at surgery was 53.1 (±8.4) years, while the mean time from diagnosis and treatment to the present study was 9.4 (±6.5) years, ranging from 3 to 18 years. Almost 35% of women were 3 to 5 years after mastectomy, and the remaining participants were over 5 years after surgery and treatment. In most of the women (63.6%), breast cancer was diagnosed after menopause. Artificial menopause caused by surgery and breast cancer treatment occurred in 36.4% of them. All patients had been treated with surgery. The vast majority of the breast cancer patients (84.8%) underwent a radical mastectomy; the remaining 15.2% underwent partial mastectomy. In 87.6% of patients, complementary therapy was used; 13.6% of women underwent radiotherapy, 27.2% underwent chemotherapy, and 30.8% underwent combined radiotherapy and chemotherapy; 16% of patients took solely tamoxifen, and 44.4% took tamoxifen as part of combined cancer therapy.

The associations between SF-36 scores and by sociodemographic, medical, and general health characteristics are shown in Table 3. The average overall quality of life (OQOL) for the study participants was 60.7 (±17.9), with a Physical Component Summary (PCS) of 57.5 ± 18.8 and Mental Component Summary (MCS) of 62.8 ± 19.8. Elderly participants at interview above 70 years of age scored significantly lower in physical functioning (PF) (*p* = 0.037). Women who lived in the city acquired a significantly greater mean score in physical functioning (PF) (*p* = 0.039), role physical (RP) (*p* = 0.040), social functioning (SF) (*p* = 0.028), and role emotional (RE) (*p* = 0.042) domains compared to those who lived in the village. QOL assessment was also dependent on the level of education. Bodily pain (BP) was scored lower in women with education below 12 years (*p* = 0.001) and in smoking patients (*p* = 0.048). Better education also positively affected QOL in terms of mental health (MH) (*p* = 0.018) and PCS (*p* = 0.019). As to living situation, patients living with a partner scored higher than the others in physical health (PF (*p* = 0.039), RP (*p* = 0.014), PCS (*p* = 0.028)) and in the SF domain of mental health (*p* = 0.033). Women who had the history of pregnancy had a worse result in terms of PF (*p* = 0.021), RP (*p* = 0.009), vitality (VT) (*p* = 0.048), RE (*p* = 0.029), PCS (*p* = 0.024), MCS (*p* = 0.014), and OQOL (*p* = 0.010). The type of surgery was related to mental health. Patients after breast-conserving surgery had a greater mean MH score (*p* = 0.047) compared to those who had a radical mastectomy. QOL of the participants with abdominal obesity (waist circumference ≥80 cm) was significantly lower in all SF-36 domains (*p* = 0.001–0.030) with the exception of RP and GH. The presence of comorbidities has lowered both physical and mental QOL. Patients with cardiovascular diseases obtained lower scores in all domains of the SF-36 questionnaire (*p* ≤ 0.001–0.026), as did women with depression (*p* ≤ 0.001–0.025). SF36 scores for diabetics were lower in BP (0.003), GH (<0.001), VT (*p* = 0.020), SF (*p* = 0.001), MH (*p* = 0.003), PCS (*p* = 0.007), and OQOL (*p* = 0.016). In addition, individuals who reported arthritis were associated with lower scores in domains of PF (*p* = 0.001), RP (*p* = 0.026), BP (*p* ≤ 0.001), GH (*p* = 0.003), VT (*p* = 0.029), and PCS (*p* < 0.001) and in OQOL (0.002), whereas patients with hypertension were associated with a lower score only in domain of PF (*p* = 0.005). The individuals who exercised had higher scores in domains related to physical and mental health. Physical activity had an important role in RP (*p* = 0.047), GH (*p* = 0.009), VT (*p* = 0.003), MH (*p* = 0.001), PCS (*p* = 0.032), MCS (*p* = 0.017), and OQOL (*p* = 0.046).

Finally, a linear multivariate regression analysis was used to detect any variable independently related to QOL of participants. Table 4 presents the coefficients of multivariate models of linear regression evaluating the association between sociodemographic and medical factors (explanatory variables) and the scores for each dimension of the SF-36, summary components (PCS and MCS), and overall OQOL (explained variables). Our study evidenced that depression, chronic diseases, abdominal obesity, and history of pregnancy significantly reduced PCS, MCS, and overall OQOL in women long after the completion of their breast cancer treatment. In turn, participation in regular physical activity and living with a partner were the most significant factors positively affecting PCS, MCS, and OQOL. Additionally, education level ≥ 12 years was significantly positively associated only with PCS, whilst living in the city was a significant factor affecting MCS. Age, time since surgery, type of treatment, occupational status, and smoking status were shown to have no significant impact on the QOL in women long after the completion of their breast cancer treatment. The coefficients of determination (R^2^) in the models of multivariate regression for PCS, MCS, and OQOL were 0.23, 0.32, and 0.34, respectively. This means that 23% of PCS variation, 32% of MCS variation, and 34% of OQOL variation are explained by variables in particular models. The fitting of the models was therefore not satisfactory, albeit statistically significant.

## 4. Discussion

QOL assessment is an important aspect of the health care of oncological patients, and it should be complementary to medical procedures. This study assesses which factors affected the QOL of Polish postmastectomy women long after breast cancer diagnosis and treatment. Breast cancer and mastectomy have a negative impact on QOL, causing long-term disturbances in mental and physical functioning [2,3,19]. Low QOL assessment in these domains is caused by cancer diagnosis and the uncertainty of treatment outcomes. Currently available results indicate that the QOL assessment in women with diagnosed breast cancer changes, depending on the stage of treatment and medical interventions.

Generally, lower QOL after breast cancer diagnosis was found in younger women who were significantly more exposed to cancer-related depression, anxiety, and stress [20]. Many authors found much worse QOL in its physical functioning components in patients after surgery than before the commencement of breast cancer treatment [21]. Irritability and depression with significantly worse emotional and social functioning frequently occurred [3]. The results of a long-term observation for 12 months after the beginning of the therapy revealed a significant improvement in most QOL subscales and return to baseline [20]. As discussed by Jones et al. [22], breast cancer patients still experienced depression symptoms and worsened physical fitness and mental health status one year after cancer diagnosis; however, the intensity of these symptoms was lower than that at six months after the diagnosis. In another study, the patients reported problems with global QOL, pain, and body image 18 months after the commencement of therapy [19]. A 24-month observation of breast cancer patients undergoing surgery revealed an improvement in physical, cognitive, emotional, and social functioning, as well as in general health status [23].

In the present study, the mean time after diagnosis in the group of women after mastectomy was more than 9 years. The mean score of OQOL of the subjects was 60.7 (±17.9), at the level of relatively good. Patients had better performance in the MCS (62.8 ± 19.8) component and weaker performance in the PCS (57.5 ± 18.8) component. The results were comparable or better than the norm for healthy Polish women aged 50 to 60 years [24,25]. The multivariate regression analysis revealed no relationship between the time since surgery and patients’ QOL. Age was also not a significant predictor of the PCS, MCS, and OQOL, though the women over 69 years of age had a lower QOL assessment in terms of physical functioning (PF). Dorval et al. [26] compared the QOL of women eight years after breast cancer diagnosis with a group of women without breast cancer. They found similar QOL assessments in both groups with the exception of problems with arm function and lowered sexual satisfaction in women after breast cancer treatment, who lived with a partner. However, women who experienced breast cancer recurrence revealed lower QOL assessment in all areas except social functioning [26]. Long-term cancer survivors, 12.5 years after diagnosis, had a similar QOL assessment to the controls, experiencing slightly worse cognitive functioning and financial status [27]. Jones et al. [22], in their study of long-term consequences of breast cancer diagnosis, showed that depression symptoms in breast cancer patients returned to baseline 10 years after diagnosis; however, their physical fitness and mental health levels were still significantly lower even 10 years after diagnosis. Another study found that breast cancer survivors, on average 13 years after initial diagnosis, had lower mental health scores compared to the general population [28].

A significant determinant of QOL of breast cancer patients is the type of used therapy. Mols et al. [29] studied the QOL of breast cancer patients who survived for at least five years after diagnosis. They showed that the current medical disease status, social support, and level of income were strong positive predictors of QOL, whereas undergoing complementary chemotherapy was a negative predictor. Ganz et al. [30] found that patients who survived 5–10 years after the initial diagnosis had a high assessment of QOL; however, those who had never undergone a systemic complementary therapy had a higher QOL in terms of physical functioning, bodily pain, social functioning, and general health than those who had received chemotherapy, tamoxifen, or combined therapy. The results of the present study did not reveal long-term effects of lower QOL dependent on complementary therapy. The multivariate regression models of using radiotherapy, chemotherapy, and tamoxifen revealed no significant impact. Engel et al. [31] found that complementary therapy was not a significant predictor of QoL in breast cancer patients. We argue that the type of used adjuvant therapy does not have an influence on the QOL of women long after completion of breast cancer treatment.

A significant determinant of QOL of breast cancer patients is the type of surgery. In our study, the type of surgery was not an independent predictor of the quality of life of the studied women. However, women after radical mastectomy had statistically significantly worse mental health compared to those who underwent conservative surgery. Different studies have reported conflicting results regarding the QOL scores of these two groups of patients. Mostly the QOL scores of the partial mastectomy group were better than those of the radical mastectomy group, especially in the domain of mental health [32,33,34].

A significant predictor of low QOL in breast cancer patients is the occurrence of comorbidities. The current study revealed the effects of comorbidities on the lowering of QOL in all SF-36 subscales, PCS, MCS, and OQOL. The occurrence of cardiovascular diseases, hypertension, diabetes, arthritis, and depression reduced the quality of physical and mental health, including social function. Only thyroid disease, which occurred in almost 25% of women, had no impact on their quality of life in any domain. Similar results were found among breast cancer patients by Fu et al. [8], in whose study the comorbidities, in particular, hypertension, arthritis, and diabetes were negatively correlated with QOL components in terms of general health, physical functioning, bodily pain, and vitality. Kearns et al. [35] revealed a lower QOL in individuals with body mass index (BMI) above normal and proved that diabetes, heart disease, arthritis, and hypertension may mediate the relationship between overweight and QOL. Obesity, often found in breast cancer patients, is one of the proven risk factors for breast cancer and a potential post-treatment complication [6].

Low physical activity and tendencies towards overweight and obesity in breast cancer patients are usually associated with lower QOL [4,5,6,7]. The results of several studies indicate that not only BMI, but rather a larger waist circumference, which is more strongly correlated with the amount of visceral fat, is independently and positively associated with breast cancer risk in both the premenopausal and postmenopausal periods [36,37]. In addition, in studies of different groups of healthy subjects and patients, multivariate models showed a tendency for waist circumference to be significantly superior to other adipometric variables, including waist–hip ratio (WHR), whose usefulness was found to be lower [37,38,39,40]. Thus, waist circumference is a better predictor of health risks, and relations between excessive central fatness and QOL have been discussed by many authors, especially with regard to comorbidities and physical functioning disorders [41]. In the current study, visceral fatness, as measured by waist circumference, occurred in 88.4% of women and was negatively correlated with QOL assessment. Women with a larger waist circumference had worse levels of physical, mental, and social functioning. Central adiposity was an independent predictor of PCS, MCS, and OQOL.

The group of factors that lowered the QOL of breast cancer patients in the present study also included loneliness, pregnancy history, and living in a rural area. Among the examined variables in the present study, loneliness impaired physical health and social functioning and was identified as an independent predictor for the PCS, MCS, and OQOL. These results correspond to those in other studies, which revealed that loneliness is a strong predictor of mental suffering and physical problems in breast cancer patients [15,42]. Family and social support in breast cancer patients undergoing surgery was the main cause of improvement in most QOL subscales, mainly in the components of mental and social functioning [20,23].

The results of this study indicate that another factor that significantly influenced QOL was pregnancy history. We found that pregnancy history lowered physical and mental health and was an independent predictor of PCS, MCS, and OQOL. Other studies showed a rather significant positive relationship between QOL score and having children [43]. Many factors may influence the differences in QOL scores between women with a history of pregnancy and those who have not given birth. Research suggests that pregnancy and motherhood can have long-lasting effects on maternal health. Factors that may lower the QOL of women with a reproductive history include the stress of long-term childcare, providing material support to children, a higher prevalence of joint pain, and a tendency toward obesity [44,45,46].

According to the results of our study, breast cancer survivors who lived in the city acquired a significantly greater mean score in emotional, physical, and social functioning compared to those who lived in the village. Living in the city was a positive and independent predictor of MCS. A recent QOL study of a population of Polish women living in rural areas found that 42% of the surveyed women assessed their standard of living as average, 52% did not see the advantages of living in the countryside, and 70% of the respondents declared a desire to migrate to the city. The share of women defining their income as low was 44% [47]. Previous research on the breast cancer population from central and eastern Poland revealed a statistically significant correlation between place of residence and the social component of QOL; i.e., higher QOL assessments were found in rural than in urban women [48]. Other studies also estimated that rural breast cancer survivors had a statistically significantly higher overall QoL and experienced a lower symptom burden than urban survivors. These authors suggest that quality of life does not depend solely on access to services and professionals, but may include individual- and community-level psychosocial factors [49]. We believe that the observed differences between various regions of Poland may result from different socioeconomic and family conditions.

In the present study, only physical activity, education level, living with a partner, and living in the city were significant predictors of QOL improvement. The women after breast cancer treatment with a secondary education (12 years of education) or higher education (more than 12 years of education) featured better QOL assessments in the physical functioning area (BP, PCS) and MH. Education level was an independent predictor of PCS. A number of studies have shown a correlation between education level and higher health awareness, healthier lifestyles, and lower tendency towards addictions. In addition, higher education correlates with higher income. All of these factors can influence QOL. Sociodemographic characteristics such as age, education level, and monthly household revenue affected the QOL of breast cancer patients and healthy women [15,50]. The overall QOL of postmastectomy women is generally high, especially among women with a higher level of education, whereas a lower level of education was a predictor of low QOL [48]. Among healthy Polish postmenopausal women from urban and rural communities, educational attainment and employment status were the most powerful independent risk factors for health-related QOL [51]. In our study, employment was not correlated with the QOL long-term survivors of breast cancer in any domain of SF-36.

Many studies show the negative impact of smoking on quality of life of patients with cancer. In the present study, smoking was an independent negative predictor of vitality in the area of mental health. We also showed that the perception of pain was greater in smokers compared to nonsmokers. Such a relationship has been demonstrated in other studies. In patients with cancer, also with breast cancer, smoking status had a main effect and an interaction with time upon depression and pain severity [52].

Positive effects of physical activity on body composition and QOL indices in breast cancer patients have been indicated by many authors [7,53]. In the present study, regular physical activity improved general health, vitality, and physical and mental health and was an independent predictor of PCS, MCS, and OQOL. We agree with the assertion that the very presence of the disease lowers the QOL in breast cancer patients [1] and that body mass disorders, loneliness, and place of residence may deteriorate physical and mental functioning. In conclusion, women who were from the city; were educated; never gave birth; were without abdominal obesity, depression, and other comorbidities; were living with a partner; and were undertaking physical activity had better overall QOL.

The regression models created in our study for particular SF-36 subscales in the group of women after mastectomy explained a maximum of 35.6% of the variance. This shows that QOL is determined by multiple factors and that not all significant explanatory variables might have been considered in the present study.

### Limitations

This study has several limitations. The results of our study show the level of QOL in women after mastectomy participating in meetings of associations whose mission is to support psychologically and enable participation in organized physical activity. Recent Polish studies have shown that membership in breast cancer self-help groups improves the functioning of breast cancer patients in a five-year period after treatment completion [54]. The Federation of Amazon Associations brings together about 26,000 patients in 211 Amazon clubs in Poland [55]. A Polish study conducted by Pacian et al. [56] shows that women with higher and secondary education (85%), living in cities (91.7%) where these clubs operate, seek help in an Amazon club. Similar results are provided by our study: 71.6% and 90.4%, respectively (Table 1). Women with breast cancer seek support in their immediate locality, which reduces costs and time spent on travel [56]. Therefore, the results of our study may not be representative of the general population of breast cancer patients. Despite this limitation, this study provides a more in-depth representation of one setting, postmastectomy clubs, which have been active in Poland for 28 years. Future studies into QOL should cover women who completed their breast cancer therapy but do not make use of mutual support groups.

Another limitation of the current study is the lack of detailed clinical data such as type and stage of cancer. We did not have access to hospital cards; the study participants answered the questions alone, so we limited the interview to the simplest questions.

Furthermore, we did not include a breast reconstruction element in our research, which is another limitation of the study. A recent meta-analysis of the current literature has shown that breast-conserving surgery, compared with total mastectomy, leads to better outcomes in terms of body image, future perspectives, and less systemic side effects [57]. A Polish study of women with breast cancer from the same region of Poland as the participants of our study showed that patients treated surgically with simultaneous breast reconstruction rated QOL better only in the domains of sexual functioning and satisfaction with intercourse with a partner. No differences were found in the other domains of QOL compared to women who underwent mastectomy without breast reconstruction. In addition, patients who opted for breast reconstruction were statistically significantly younger (mean 48.9 years) compared to those after mastectomy (mean 56.5 years) [58]. In our study, 66.8% were women aged over 50 years at the time of surgery.

Another possible limitation is the lack of information on income, which can have a significant impact on QOL. Only a few women chose to answer this question, and therefore we did not include this variable in the data analysis. Furthermore, we believe that future studies should include a comparable group of women without breast cancer to determine whether QOL and related factors are similar among women with and without breast cancer.

## 5. Conclusions

Health education, greater social support, specialist care in the treatment of comorbidities, and propagation of a physically active lifestyle can improve the physical and mental functioning of breast cancer survivors long after diagnosis and treatment.

## Figures and Tables

**Table 1:** Sociodemographic and general health characteristics of the participants (*n* = 250).

Variables	Mean ± SD No. (%)
Age (years)	62.8 ± 8.0
<50	14 (5.6)
50–69	192 (76.8)
≥70	44 (17.6)
Place of residence	
Village	24 (9.6)
City	226 (90.4)
Education level	
<12 years	71 (28.4)
≥12 years	179 (71.6)
Living situation	
With a partner	160 (64.0)
Single	90 (36.0)
Occupational status	
Working	26 (10.4)
Nonworking	224 (89.6)
Pregnancy history	
No	28 (11.2)
Yes	222 (88.2)
Smoking status	
No	138 (55.2)
Yes	112 (44.8)
Waist circumference (cm)	108.8 ± 10.6
<80	29 (11.6)
≥80	221 (88.4)
Types of chronic diseases Diabetes	
No	215 (86.0)
Yes	35 (14.0)
Hypertension	
No	148 (59.2)
Yes	102 (40.8)
Cardiovascular disease	
No	197 (78.8)
Yes	53 (21.2)
Arthritis	
No	157 (62.8)
Yes	93 (37.2)
Thyroid disease	
No	188 (75.2)
Yes	62 (24.8)
Depression	
No	228 (91.2)
Yes	22 (8.8)
Other diseases	
No	217 (86.8)
Yes	33 (13.2)
Physical activity	
No	147 (58.8)
Yes	103 (41.2)

**Table 2:** Clinical data of breast cancer survivors (*n* = 250).

Variables	Mean ± SD No. (%)
Age at surgery (years) Range 3–18 years	53.1 ± 8.4
Time since surgery (years)	9.4 ± 6.5
≤5	87 (34.8)
>5	163 (65.2)
Menopause	
Natural	159 (63.6)
Artificial	91 (36.4)
Surgical treatment	
Radical mastectomy	212 (84.8)
Partial mastectomy	38 (15.2)
Complementary therapy Radiotherapy	219 (87.6) 34 (13.6)
Chemotherapy	68 (27.2)
Radiotherapy and chemotherapy	77 (30.8)
Tamoxifen	
Taken solely	40 (16.0)
Part of combined therapy	111 (44.4)

**Table 3:** SF-36 scores by sociodemographic, medical, and general health characteristics in long-term breast cancer survivors (*n* = 250).

Variables	PF	RP	BP	GH	VT	SF	RE	MH	PCS	MCS	OQOL
Total participants	63.5 (20.5)	57.2 (35.1)	54.8 (24.5)	53.2 (18.8)	53.9 (19.7)	69.5 (24.0)	62.7 (35)	61.7 (18.4)	57.5 (18.8)	62.8 (19.8)	60.7 (17.9)
Age (years)											
<50	67.3 (17.9)	54.2 (41.6)	54.1 (24.0)	52.5 (22.2)	52.8 (22.1)	70.8 (25.7)	58.3 (44.7)	60.1 (20.6)	57.0 (17.3)	64.3 (22.0)	62.1 (15.9)
50–69	64.7 (20.1)	58.7 (33.5)	53.5 (24.3)	53.4 (19.1)	54.6 (19.7)	69.9 (24.1)	62.0 (34.6)	61.7 (18.0)	57.7 (18.7)	62.6 (19.8)	60.4 (18.0)
≥70	56.9 (22.1)	49.7 (40.8)	60.2 (25.6)	53.0 (17.1)	51.3 (18.7)	67.8 (24.0)	67.5 (34.4)	62.5 (20.3)	56.1 (20.3)	61.3 (20.2)	61.2 (18.8)
*p* value	0.037 *	0.290	0.209	0.982	0.580	0.834	0.580	0.909	0.887	0.942	0.927
Place of residence											
Village	55.4 (25.8)	40.8 (33.1)	46.6 (24.5)	51.1 (17.1)	47.1 (25.3)	59.2 (20.3)	44.1 (39.6)	54.8 (23.1)	51.7 (18.8)	53.9 (24.3)	53.8 (20.4)
City	65.1 (19.2)	59.3 (36.1)	56.2 (24.4)	53.7 (19.0)	54.8 (19.2)	71.7 (23.8)	64.3 (35.8)	62.8 (17.7)	58.7 (18.7)	63.8 (19.8)	61.7 (17.8)
*p* value	0.039 *	0.040 *	0.053	0.565	0.123	0.028 *	0.042 *	0.082	0.078	0.080	0.074
Education level (years)											
<12	59.9 (21.9)	52.9 (32.8)	47.3 (21.5)	52.4 (15.9)	49.6 (20.1)	67.7 (25.3)	57.9 (33.9)	56.8 (18.4)	52.7 (16.9)	58.7 (18.9)	56.3 (16.2)
≥12	65.0 (20.0)	59.1 (35.7)	57.8 (25.0)	53.9 (19.8)	55.4 (19.2)	70.0 (23.7)	64.3 (35.4)	63.4 (18.3)	59.5 (19.2)	64.1 (20.1)	62.3 (18.4)
*p* value	0.063	0.191	0.001 *	0.532	0.110	0.479	0.179	0.018 *	0.019 *	0.059	0.054
Living situation											
With a partner	65.4 (19.4)	61.3 (33.8)	56.6 (23.5)	54.1 (18.3)	55.5 (18.8)	71.8 (23.3)	63.5 (35.2)	62.0 (17.8)	59.3 (17.7)	63.7 (19.3)	62.0 (17.2)
Alone	60.3 (22.5)	50.5 (35.8)	52.2 (26.3)	52.8 (19.7)	51.1 (21.4)	65.5 (25.3)	61.6 (34.6)	61.6 (19.9)	54.8 (20.6)	61.3 (21.0)	58.6 (19.4)
*p* value	0.039 *	0.014 *	0.140	0.571	0.076	0.033 *	0.668	0.873	0.028 *	0.354	0.159
Occupational status											
Working	70.3 (16.9)	64.5 (35.5)	63.5 (29.0)	59.1 (17.8)	56.9 (17.0)	76.9 (19.8)	68.9 (37.9)	62.9 (18.0)	64.3 (17.6)	66.8 (20.7)	65.5 (17.6)
Nonworking	63.8 (20.1)	57.8 (36.0)	54.9 (23.9)	53.5 (18.6)	54.3 (20.3)	70.2 (24.3)	63.2 (35.7)	62.4 (18.5)	57.8 (18.7)	63.2 (20.2)	61.1 (18.0)
*p* value	0.105	0.393	0.094	0.135	0.397	0.213	0.326	0.820	0.064	0.335	0.174
Pregnancy history											
No	72.6 (18.9)	75.1 (24.9)	59.4 (27.2)	54.0 (20.1)	61.6 (19.7)	75.0 (25.5)	78.4 (24.9)	63.6 (21.2)	66.0 (18.9)	72.9 (19.0)	70.3 (17.9)
Yes	62.6 (20.5)	55.7 (35.2)	54.4 (24.1)	53.6 (18.4)	53.2 (19.7)	69.3 (23.9)	61.5 (35.4)	61.4 (18.2)	56.8 (18.5)	62.0 (19.7)	60.0 (17.6)
*p* value	0.021 *	0.009 *	0.330	0.929	0.048 *	0.265	0.029 *	0.574	0.024 *	0.014 *	0.010 *
Surgical treatment											
Partial mastectomy	66.6 (20.9)	59.2 (36.6)	57.3 (27.9)	57.4 (20.7)	57.9 (20.9)	72.2 (22.6)	64.0 (38.1)	66.8 (16.9)	60.9 (19.7)	66.1 (19.8)	64.0 (18.8)
Radical mastectomy	63.4 (20.1)	57.8 (34.9)	54.1 (23.6)	52.8 (18.3)	52.8 (19.2)	68.8 (24.3)	62.9 (34.6)	60.6 (18.2)	57.2 (18.5)	62.3 (19.8)	60.2 (17.7)
*p* value	0.343	0.815	0.422	0.127	0.114	0.389	0.858	0.047 *	0.229	0.261	0.212
Radiotherapy											
No	61.8 (21.4)	58.5 (35.7)	54.2 (23.9)	52.8 (19.8)	52.7 (18.5)	69.1 (23.0)	63.8 (34.3)	60.4 (18.6)	57.1 (18.7)	62.5 (19.9)	60.47 (17.9)
Yes	65.3 (19.6)	56.7 (34.3)	54.9 (25.0)	54.0 (17.9)	53.8 (20.1)	70.1 (23.5)	61.0 (35.9)	62.5 (17.2)	58.1 (18.7)	62.4 (20.0)	60.74 (18.0)
*p* value	0.155	0.680	0.823	0.574	0.660	0.719	0.530	0.348	0.674	0.991	0.909
Chemotherapy											
No	62.9 (21.5)	57.6 (34.1)	56.4 (25.0)	51.4 (17.2)	55.6 (17.0)	69.6 (22.6)	62.8 (34.6)	63.1 (16.7)	57.5 (18.3)	63.7 (17.8)	61.3 (16.8)
Yes	63.7 (20.1)	57.7 (35.6)	53.4 (24.0)	54.6 (19.6)	51.8 (20.4)	69.5 (23.7)	62.3 (35.4)	60.3 (18.6)	57.6 (18.9)	61.8 (21.1)	60.2 (18.6)
*p* value	0.741	0.985	0.315	0.172	0.113	0.985	0.907	0.227	0.969	0.476	0.655
Tamoxifen											
No	63.6 (21.4)	57.5 (34.2)	57.4 (24.5)	54.4 (19.2)	52.2 (22.1)	72.5 (23.9)	66.2 (34.4)	59.2 (20.4)	58.6 (19.5)	63.5 (21.8)	61.1 (19.1)
Yes	63.2 (19.9)	56.8 (35.5)	52.7 (24.3)	52.4 (18.4)	54.0 (17.4)	68.6 (22.5)	60.4 (35.1)	62.3 (16.5)	56.6 (18.1)	62.0 (18.4)	60.0 (17.0)
*p* value	0.889	0.858	0.113	0.363	0.451	0.167	0.178	0.179	0.370	0.572	0.637
Time since surgery (years)											
≤5	63.4 (19.2)	52.6 (36.6)	55.5 (23.8)	53.9 (19.3)	53.2 (18.8)	69.3 (24.8)	59.9 (35.5)	60.1 (20.0)	56.5 (17.8)	61.2 (20.9)	59.2 (18.0)
>5	63.5 (21.2)	59.8 (34.2)	53.8 (24.7)	53.0 (18.7)	53.9 (20.1)	70.9 (22.3)	64.0 (34.9)	62.2 (17.5)	57.8 (19.4)	63.5 (19.4)	61.2 (18.1)
*p* value	0.972	0.114	0.566	0.729	0.780	0.578	0.371	0.402	0.612	0.391	0.412
Waist circumference (cm)											
<80	75.1 (13.9)	69.0 (36.3)	65.6 (25.9)	58.5 (20.0)	63.9 (19.1)	79.3 (21.7)	80.1 (31.0)	70.0 (21.9)	66.9 (19.2)	72.4 (20.6)	69.1 (17.6)
≥80	62.9 (20.0)	56.4 (36.1)	54.0 (23.9)	52.5 (18.5)	53.1 (19.7)	69.1 (24.4)	61.0 (36.2)	61.5 (17.6)	56.8 (18.4)	62.0 (19.7)	59.9 (17.7)
*p* value	0.001 *	0.077	0.023 *	0.221	0.006 *	0.030 *	0.008 *	0.020 *	0.010 *	0.006 *	0.011 *
Smoking											
No	63.7 (21.1)	56.7 (35.1)	57.2 (25.4)	51.6 (18.6)	56.6 (20.7)	68.2 (22.9)	61.3 (35.5)	62.1 (19.4)	58.6 (19.5)	62.2 (19.2)	61.0 (17.5)
Yes	63.2 (20.5)	58.8 (34.7)	51.6 (22.1)	55.5 (19.0)	50.7 (19.8)	70.6 (25.5)	64.3 (34.4)	61.2 (17.9)	55.3 (18.6)	60.8 (21.1)	58.7 (18.6)
*p* value	0.844	0.662	0.048 *	0.083	0.050	0.411	0.486	0.713	0.255	0.677	0.414
Diabetes											
No	64.4 (20.5)	58.7 (34.9)	56.8 (24.1)	55.6 (18.2)	55.1 (19.8)	71.5 (23.1)	63.9 (35.4)	63.1 (18.0)	59.3 (18.3)	63.9 (20.0)	62.1 (17.8)
Yes	58.6 (20.1)	48.3 (34.5)	44.2 (24.7)	43.8 (17.8)	46.8 (20.1)	58.0 (27.6)	55.0 (33.7)	53.1 (21.4)	48.6 (18.9)	56.5 (19.7)	53.3 (17.6)
*p* value	0.108	0.101	0.003 *	<0.001 *	0.020 *	0.001 *	0.165	0.003 *	0.007 *	0.052	0.016 *
Hypertension											
No	66.4 (19.2)	57.5 (35.5)	55.8 (25.3)	55.4 (19.5)	54.7 (20.4)	70.1 (24.4)	61.1 (36.0)	61.8 (18.4)	59.0 (18.9)	62.3 (20.4)	61.1 (18.3)
Yes	59.4 (21.8)	57.0 (34.2)	53.9 (23.4)	51.9 (16.8)	52.9 (19.5)	68.9 (23.8)	64.9 (34.0)	61.7 (19.5)	56.0 (18.3)	63.8 (19.6)	60.7 (17.5)
*p* value	0.005 *	0.909	0.520	0.114	0.492	0.679	0.399	0.973	0.203	0.582	0.857
Cardiovascular disease											
No	65.4 (19.6)	60.1 (35.1)	57.5 (24.4)	55.5 (18.8)	56.0 (19.7)	71.3 (24.0)	65.5 (35.0)	63.3 (18.9)	60.1 (18.3)	64.6 (20.3)	62.8 (18.1)
Yes	56.7 (22.5)	47.4 (32.7)	46.0 (23.1)	48.5 (16.5)	46.7 (19.5)	63.5 (23.7)	52.5 (34.0)	55.8 (17.4)	49.7 (17.9)	56.7 (17.9)	54.1 (15.8)
*p* value	0.003 *	0.014 *	0.001 *	0.009 *	0.001 *	0.026 *	0.013 *	0.009 *	<0.001 *	0.012 *	0.002 *
Arthritis											
No	66.5 (19.8)	61.0 (34.5)	58.8 (23.9)	56.5 (18.3)	56.0 (19.0)	71.6 (23.9)	63.6 (36.3)	62.8 (17.2)	61.1 (17.3)	64.9 (18.8)	63.7 (19.6)
Yes	58.5 (20.8)	51.2 (34.9)	48.7 (24.4)	49.7 (18.1)	50.5 (21.3)	66.1 (24.2)	61.2 (33.4)	60.0 (21.3)	52.3 (19.7)	59.6 (21.7)	56.3 (16.4)
*p* value	0.001 *	0.026 *	<0.001 *	0.003 *	0.029 *	0.064	0.593	0.258	<0.001 *	0.051	0.002 *
Thyroid disease											
No	62.7 (21.2)	58.2 (36.0)	55.1 (24.8)	54.8 (18.2)	54.3 (27.0)	69.2 (24.2)	61.1 (36.6)	62.1 (18.3)	58.0 (18.8)	63.0 (20.1)	61.3 (18.0)
Yes	66.0 (18.4)	54.6 (31.4)	54.6 (23.7)	51.4 (19.2)	52.9 (18.0)	77.0 (23.9)	67.4 (30.1)	60.6 (20.3)	57.1 (18.4)	62.7 (20.2)	59.7 (17.9)
*p* value	0.236	0.461	0.887	0.189	0.614	0.649	0.211	0.579	0.737	0.924	0.548
Depression											
No	64.4 (20.4)	58.9 (34.6)	56.7 (24.4)	55.0 (18.0)	55.5 (19.6)	71.5 (23.5)	65.7 (34.4)	63.5 (17.5)	59.1 (18.4)	65.3 (18.7)	63.0 (17.0)
Yes	54.7 (20.0)	41.2 (34.4)	37.6 (18.0)	44.0 (20.8)	39.5 (18.2)	50.5 (21.8)	33.0 (28.5)	44.6 (22.3)	44.6 (16.5)	41.0 (19.0)	42.2 (16.2)
*p* value	0.025 *	0.018 *	<0.001 *	0.005 *	<0.001 *	<0.001 *	<0.001 *	<0.001 *	0.001 *	<0.001 *	<0.001 *
Physical activity											
No	62.2 (21.0)	53.7 (32.3)	53.8 (25.1)	51.6 (18.3)	50.6 (20.0)	67.3 (24.5)	59.5 (32.6)	58.0 (18.6)	55.6 (18.6)	60.0 (19.5)	58.7 (17.6)
Yes	67.2 (18.6)	62.3 (36.1)	56.4 (24.0)	57.6 (18.5)	58.1 (19.0)	73.0 (23.7)	67.6 (36.5)	65.6 (17.9)	60.6 (18.4)	66.3 (20.1)	63.5 (18.0)
*p* value	0.062	0.047 *	0.401	0.009 *	0.003 *	0.056	0.063	0.001 *	0.032 *	0.017 *	0.046 *

The data are presented as mean (± standard deviation); PF—physical functioning; RP—role physical; BP—bodily pain; GH—general health; VT—vitality; SF—social functioning; RE—role emotional; MH—mental health; PCS—Physical Component Summary; MCS—Mental Component Summary; OQOL—overall quality of life; * *p* < 0.05.

**Table 4:** Predictors of quality of life in breast cancer patients long after completed treatment (*n* = 250).

Independent Variables	Dependent Variables Standardized Regression Coefficients (Β)										
	PF	RP	BP	GH	VT	SF	RE	MH	PCS	MCS	OQOL
Age (years)	−0.11	-	0.06	-	0.05	-	-	0.04	-	0.04	0.06
*p* value	0.092	-	0.433	-	0.486	-	-	0.607	-	0.636	0.452
Place of residence											
Village (ref.)	ref.	ref.	-	-	ref.	ref.	ref.	ref.	-	ref.	ref.
City	0.16	0.06	-	-	0.22	0.14	0.14	0.17	-	0.19	0.10
*p* value	0.015 *	0.290	-	-	0.003 *	0.048 *	0.028 *	0.016 *	-	0.013 *	0.166
Education level (years)											
<12 (ref.)	-	-	ref.	-	-	-	-	ref.	ref.	ref.	ref.
≥12	-	-	0.14	-	-	-	-	0.06	0.12	−0.01	0.06
*p* value		-	0.045 *	-	-	-	-	0.376	0.049 *	0.885	0.448
Living situation											
Alone (ref.)	-	ref.	ref.	-	ref.	ref.	-	-	ref.	ref.	ref.
With partner	-	0.16	0.14	-	0.20	0.039 *	-	-	0.024 *	0.049 *	0.048 *
*p* value	-	0.011 *	0.046 *	-	0.007 *	0.039 *	-	-	0.024 *	0.049 *	0.048 *
Occupational status											
Working (ref.)	-	-	-	ref.	ref.	ref.	ref.	-	-	-	-
Nonworking	-	-	-	−0.03	−0.04	−0.04	−0.01	-	-	-	-
*p* value	-	-	-	0.673	0.562	0.562	0.962	-	-	-	-
Pregnancy history											
No (ref.)	-	ref.	-	-	ref.	ref.	ref.	-	ref.	ref.	ref.
Yes	−0.14	−0.17	-	-	−0.15	−0.05	−0.13	-	−0.17	0.047 *	−0.17
*p* value	0.031 *	0.007 *	-	-	0.042 *	0.489	0.043 *	-	0.006 *	0.047 *	0.014 *
Surgical treatment											
Partial mastectomy (ref.)	ref.	-	-	-	ref.	ref.	ref.	-	-	ref.	-
Radical mastectomy	0.01	-	-	-	−0.05	0.06	0.02	-	-	0.01	-
*p* value	0.896	-	-	-	0.496	0.399	0.794	-	-	0.910	-
Complementary therapy											
No (ref.)	-	-	-	-	ref.	-	ref.	-	-	ref.	-
Yes	-	-	-	-	−0.12	-	0.02	-	-	−0.03	-
*p* value	-	-	-	-	0.122	-	0.746	-	-	0.678	-
Time since surgery (years)	-	0.10	-	-	-	-	0.01	-	-	−0.02	-
*p* value	-	0.114	-	-	-	-	0.921	-	-	0.831	-
Abdominal obesity											
No (ref.)	ref.	-	ref.	ref.	ref.	ref.	ref.	ref.	ref.	ref.	ref.
Yes	−0.14	-	−0.15	−0.15	−0.14	−0.06	−0.13	−0.16	−0.12	−0.16	−0.16
*p* value	0.029 *	-	0.027 *	0.047 *	0.045 *	0.385	0.055	0.031 *	0.048 *	0.042 *	0.026 *
Smoking status											
No (ref.)	-	-	ref.	ref.	ref.	ref.	ref.	ref.	-	ref.	ref.
Yes	-	-	−0.06	0.02	−0.15	−0.01	−0.04	−0.04	-	−0.04	−0.04
*p* value	-	-	0.347	0.832	0.033 *	0.932	0.564	0.553	-	0.572	0.588
Chronic diseases											
No (ref.)	ref.	ref.	ref.	ref.	ref.	ref.	ref.	ref.	ref.	ref.	ref.
Yes	−0.15	−0.15	−0.19	−0.23	−0.28	−0.14	−0.04	−0.18	−0.15	−0.16	−0.23
p value	0.022 *	0.015 *	0.007 *	0.002 *	<0.001 *	0.042 *	0.601	0.018 *	0.015 *	0.045 *	0.003 *
Depression											
No (ref.)	ref.	ref.	ref.	ref.	ref.	ref.	ref.	ref.	ref.	ref.	ref.
Yes	−0.22	−0.19	−0.27	−0.24	−0.30	−0.35	−0.33	−0.37	−0.30	−0.43	−0.39
p value	0.001 *	0.002 *	<0.001 *	0.001 *	<0.001 *	<0.001 *	<0.001 *	<0.001 *	<0.001 *	<0.001 *	<0.001 *
Physical activity											
No (ref.)	ref.	ref.	ref.	ref.	ref.	-	-	ref.	ref.	ref.	ref.
Yes	0.14	0.16	0.06	0.21	0.32	-	-	0.27	0.17	0.18	0.19
*p* value	0.030 *	0.008 *	0.390	0.004 *	<0.001 *	-	-	0.001 *	0.007 *	0.031 *	0.013 *
R^2^	0.182	0.132	0.192	0.181	0.356	0.205	0.183	0.300	0.233	0.323	0.336
F	5.86	5.09	5.37	5.86	6.45	4.92	6.43	6.70	8.90	5.25	7.20
*p*	<0.001	<0.001	<0.001	<0.001	<0.001	<0.001	<0.001	<0.001	<0.001	<0.001	<0.001

Reference. PF—physical functioning; RP—role physical; BP—bodily pain; GH—general health; VT—vitality; SF—social functioning; RE—role emotional; MH—mental health; PCS—Physical Component Summary; MCS—Mental Component Summary; OQOL—overall quality of life; chronic diseases—diabetes and/or hypertension and/or cardiovascular disease and/or arthritis; R^2^—coefficient of determination; F— test statistic; “-“ means the factor was not included in the regression model; * *p* < 0.05.

## Data Availability

Data confirming the reported results can be found at the Faculty of Physiotherapy of the Academy of Physical Education in Wrocław, al. Ignacego Jana Paderewskiego 35, building no. P4.
